# Comparative real-world effectiveness and safety of biologics and JAK inhibitors in atopic dermatitis: short- and medium-to-long-term analysis from a regional healthcare network in southern Spain

**DOI:** 10.3389/fmed.2025.1658843

**Published:** 2025-10-24

**Authors:** Alvaro Prados-Carmona, Francisco José Navarro Triviño, Husein Husein-ElAhmed, Ricardo Ruiz-Villaverde

**Affiliations:** ^1^Department of Dermatology, Hospital Universitario San Cecilio, Granada, Spain; ^2^Instituto Biosanitario de Granada, Ibs, Granada, Spain; ^3^Escuela Internacional de Posgrado, Universidad de Granada, Granada, Spain; ^4^Unit of Contact Eczema and Immunoallergic Diseases, Department of Dermatology, Hospital Universitario San Cecilio, Granada, Spain; ^5^Department of Medicine, University of Granada, Granada, Spain; ^6^Department of Dermatology, Hospital General de Baza, Baza, Spain

**Keywords:** advanced systemic therapies, biologic drugs, JAK inhibitors, treatment, real-world data, atopic dermatitis, comparative analysis

## Abstract

**Background:**

Biologics and Janus kinase (JAK) inhibitors have expanded treatment options for moderate-to-severe atopic dermatitis (AD), but comparative real-world data remain limited.

**Objective:**

This study aims to compare disease-related characteristics and outcomes of patients with moderate-to-severe AD treated with biologics or JAK inhibitors (JAKi) in routine clinical practice.

**Materials and methods:**

An ambispective, observational study was conducted across three hospitals in southern Spain. Patients who initiated advanced systemic therapy (AST) between 2019 and 2024 were included in the study. Baseline disease-related variables, clinical severity scores, treatment outcomes, adverse events, and drug survival were assessed. Short-term (16 weeks) and medium-to-long-term (24 and 52 weeks) intervals were considered. Super-responder rates, based on a proposed definition, were also evaluated.

**Results:**

A total of 202 patients were included (134 treated with biologics and 68 with JAK inhibitors). JAK inhibitors produced faster early responses, while biologics achieved greater absolute improvements between weeks 16 and 52, along with longer treatment persistence. The adverse event profiles differed between the drug classes: infections and acne were more common with JAKi, while ocular symptoms were more common with biologics. The difference in super-responder rates between groups was not statistically significant (16.42% vs. 23.53%, *p* = 0.25443).

**Conclusion:**

Both treatment classes were effective and well-tolerated. Differences in response dynamics and safety profiles support individualized treatment decisions.

## Highlights

- This real-world study from southern Spain compared biologics and JAK inhibitors in moderate-to-severe atopic dermatitis, using two clinically and demographically comparable patient cohorts.
- Treatment outcomes were assessed at short-term (16 weeks) and medium-to-long-term (24–52 weeks) intervals, revealing that JAK inhibitors produced faster early responses, while biologics achieved greater overall improvements between weeks 16 and 52, as well as longer treatment persistence over time. Similar super-responder rates were observed across all groups.
- Both treatment classes demonstrated favorable safety profiles, with different patterns of adverse events, supporting a tailored approach to therapy selection in routine clinical practice.

## Introduction

Atopic dermatitis (AD) is a chronic, relapsing inflammatory skin disease characterized by intense pruritus, eczematous lesions, and a significant impairment in quality of life (1). Affecting up to 20% of children and 10% of adults globally (1, 2), AD exhibits considerable clinical heterogeneity, driven by genetic predisposition, environmental factors, epidermal barrier dysfunction, and immune dysregulation (3–7). Increasing evidence underscores its systemic nature, with frequent associations with both atopic (e.g., asthma, allergic rhinitis, and food allergy) and non-atopic comorbidities (e.g., cardiovascular disease, metabolic syndrome, and mental health disorders) (5, 8–20). This systemic profile has prompted a paradigm shift in the perception and management of AD—from a purely cutaneous condition to a complex, chronic inflammatory disease requiring individualized and comprehensive care (21). Acknowledging this systemic nature is crucial for accurately assessing the long-term burden of the disease, particularly in younger patients, and for mitigating the risks associated with inadequate treatment (22, 23). This consideration has become increasingly relevant as therapeutic paradigms have evolved from merely alleviating symptoms to selectively targeting the molecular mechanisms underlying disease onset and recurrence (24, 25).

The therapeutic landscape for moderate-to-severe AD has evolved considerably in recent years with the advent of targeted systemic therapies, including monoclonal antibodies such as dupilumab, tralokinumab, and lebrikizumab, as well as small-molecule Janus kinase (JAK) inhibitors, including upadacitinib, abrocitinib, and baricitinib (3, 24–30). These agents have distinct mechanisms of action: biologics selectively inhibit specific interleukins within the Th2-skewed inflammatory pathway, whereas JAK inhibitors (JAKi) interfere with broader intracellular signaling cascades across multiple immune axes (31–34). Such differences may lead to variations in clinical outcomes, onset of action, and safety profiles, making it essential to understand their respective roles in real-world clinical practice (35).

Although randomized controlled trials (RCTs) are the gold standard for assessing efficacy and safety, the generalizability of the outcomes to the diverse populations encountered in routine care requires further consideration. Real-world data (RWD) derived from unselected patient cohorts, including those with complex comorbidities, contraindications, or prior treatment exposure, provide essential complementary evidence (36). In diseases such as psoriasis, RWD has proven critical for evaluating therapeutic performance, resource allocation, and patient stratification in clinical practice (36–39). However, such evidence is still emerging in AD, where systemic therapies have become widely available more recently (3, 40, 41). We already have several comparisons of the data collected from the different clinical trials (42–45). Despite being useful in the absence of head-to-head clinical trials, indirect approaches typically offer lower precision and carry a higher risk of bias. As a result, they fall somewhere between the internal validity of randomized controlled trials and the external relevance of real-world evidence, without fully achieving the strengths of either. The current expansion of treatment options, high placebo response rates observed in RCTs (2, 46–49), and the growing demand for personalized care reinforce the need for high-quality RWD to guide clinical decisions in AD. Currently, comparative real-world analyses between biologics and JAKi in AD remain limited (50–52). Importantly, these comparisons are only meaningful if underlying baseline characteristics between treatment groups are adequately balanced, as these therapeutic classes are sometimes prescribed to patients with different clinical profiles (51–53).

In this context, our group conducted a preliminary study to evaluate the baseline demographic and clinical profiles of patients with moderate-to-severe AD who initiated treatment with either biologics or JAKi in our healthcare area in southern Spain—a region serving over 500,000 inhabitants. Our initial analysis revealed a high degree of comparability between the groups (54). The present study builds upon this baseline evaluation by incorporating additional disease-specific variables and examining real-world differences in effectiveness and safety between biologics and JAKi. We aim to provide clinically relevant evidence that supports decision-making when selecting between these two families of drugs.

## Materials and methods

Study design and setting: Observational, descriptive, ambispective study conducted across three hospitals in the same province in southern Spain: Hospital Universitario San Cecilio, Granada (serving approximately 350,000 inhabitants); Hospital Santa Ana, Motril (approximately 120,000); and Hospital de Baza (approximately 65,000). Data were collected for patients treated from January 2019 through December 2024. Patients were divided into two treatment groups: Group 1 included those receiving biologic agents (dupilumab, tralokinumab, and lebrikizumab), and Group 2 comprised those treated with JAKi (upadacitinib, abrocitinib, and baricitinib).

Study population and patient selection: The sample size was determined based on the effect sizes reported in previous comparisons between biologics and JAKi in AD. With a significance level of 0.05 and a statistical power of 0.80, the final cohort (*n* = 202) was adequate to detect clinically relevant differences in treatment outcomes between groups while minimizing the risk of both type I and type II errors. Additionally, this sample size is consistent with or exceeds that of comparable real-world studies in AD and other immune-mediated inflammatory diseases treated with advanced systemic therapies (AST). The study population included patients with a confirmed diagnosis of moderate-to-severe AD, eligible for AST at any point during the study period. Patients were identified retrospectively through hospital electronic medical records (EMR) and prospectively during routine dermatology consultations.

The inclusion criteria were as follows:

(a) Age ≥ 6 years.
(b) Diagnosis of AD based on established diagnostic criteria, including Hanifin and Rajka, the UK Working Party criteria, or validated clinical adaptations (55–59).
(c) Initiation of AST with either a biologic agent or a JAKi.
(d) Availability of sufficient baseline data for characterization and data interpretation.
(e) Provision of written informed consent for using their medical data in research (or consent from the relevant legal guardian).

The exclusion criteria were as follows:

(a) Patients who did not consent to participate in the study.
(b) Use of both biologic therapies and JAKi concurrently for any reason.
(c) AD patients receiving these therapies for off label uses unrelated to AD.
(d) Pregnancy, breastfeeding, or other known contraindications for using the drugs under study.
(e) Evidence of adherence issues with previous treatments, as assessed by the treating dermatologist.

Follow-up and treatment restrictions: AST was prescribed within the Spanish National Health System in accordance with national guidelines, which restrict its use to patients with moderate-to-severe AD who have failed to achieve adequate disease control with at least one prior conventional systemic treatment or have proven contraindications for its use. All eligible patients were registered on a national digital platform, ensuring compliance with access criteria and eligibility for government-funded treatment. This study was conducted under real-world clinical practice conditions, with no protocol-mandated intensification of follow-up beyond routine care. As such, the concomitant use of topical therapies—including corticosteroids and calcineurin inhibitors—as well as overlap with conventional systemic treatments (e.g., ciclosporin, methotrexate, phototherapy) was permitted at the discretion of the treating physician, according to individual patient needs. The only restriction, as per exclusion criteria, was the concurrent use of biologics and JAKi.

### Baseline comparability

A previous pilot study ensured homogeneity between the two treatment groups regarding non-AD-related baseline characteristics: sociodemographic data, body measurements (body mass index and abdominal perimeter); lifestyle and behavioral habits (area of residence, frequency of physical exercise, alcohol and tobacco consumption); overall cardiovascular risk profiles (including cardiovascular risk factors such as diabetes mellitus, hypertension, dyslipidemia, metabolic syndrome, and chronic kidney disease); allergies and associated conditions (pneumoallergens, trophoallergens, known drug allergies, allergic contact dermatitis); as well as atopic (asthma, allergic rhinoconjunctivitis, eosinophilic oesophagitis, and nasal polyps) and other non-atopic (psoriasis, other skin conditions, ophthalmologic disease, celiac disease, gastroesophageal reflux, alopecia areata, and neoplasms) comorbidities, which were largely balanced between the groups (54). This approach ensured that neither group exhibited a higher prevalence of cardiovascular disease or atopic comorbidities in the biologic group, as could be the case in some real-world cohorts based on clinical trial data. Only minor differences were noted, such as a significantly higher rate of hypertension in patients treated with JAKi and a greater, though not statistically significant, proportion of patients under 18 years treated with biologics, in line with the chronology of regulatory approval for these drugs in adolescents and infants (54).

### Variables assessed

Data were collected across several domains. Disease-related variables included characteristics such as age of onset, disease duration, family and personal history of atopic conditions, previous treatments received, and healthcare resource use (emergency department visits and hospitalizations). Baseline disease severity was assessed using standard clinical scales, including the Eczema Area and Severity Index (EASI), Body Surface Area (BSA), Investigator’s Global Assessment (IGA), and Visual Analogue Scales (VAS) for pruritus, pain, and sleep disturbances. Short-term (16 weeks) and medium-to-long-term (24–52 weeks) intervals were considered. Outcome-related variables included treatment effectiveness, safety, patient-reported satisfaction, emergency department (ED) visits, and treatment survival. Adverse event (AE) severity was evaluated by the prescribing physician. AEs were categorized as administration reactions, dermatologic, ophthalmologic, infectious, gastrointestinal, systemic, locomotor, neurological, and other symptoms. Reported AE numbers and percentages refer to the number of patients experiencing at least on events within each AE category, rather than the number of events themselves. Reasons for treatment discontinuation were classified as follows: primary failure (insufficient disease control from treatment initiation), secondary failure (loss of previously achieved control), tolerance failure (discontinuation due to AE), patient decision (including loss to follow-up, pregnancy planning, or preference for a different route of administration), and other (mainly development of unrelated comorbidities incompatible with treatment continuation).

### Definition of composite variables

Super-responder status was defined as follows: (1) achievement of EASI90 or EASI100 at both weeks 16 and 24, (2) an IGA score of 0 or 1 at both time points, (3) a reduction in VAS pruritus of ≥4 points from baseline (provided the baseline score was >4), (4) a final VAS pruritus score of ≤2, (5) a treatment satisfaction score of ≥7, (6) no use of concomitant systemic medication during the treatment period, and (7) no use of intensified posology throughout the treatment period. This strict, multidimensional category was designed to identify patients with rapid, deep, and sustained clinical responses under real-world conditions, while ensuring that outcomes were not confounded by dose optimization or adjuvant therapies.

### Data collection and statistical analysis

Data were gathered using a combination of retrospective and prospective methods. Retrospective data were extracted from EMR. The index date was defined as the date of initiation of AST. Baseline data were defined as the closest values recorded within 6 months prior to or on the index date. Prospectively, data were collected using standardized, study-specific forms completed during dermatological consultations with informed patient participation. No imputation of missing values was performed. Baseline data were complete for all patients according to the inclusion criteria. For longitudinal effectiveness outcomes, data were analyzed on an on-treatment basis, with only values available at each scheduled time point (weeks 16, 24, and 52) included. Patients who discontinued treatment before a given time point contributed their available data up to the last visit before discontinuation but were excluded from analyses of subsequent visits. This approach was selected to prevent artificially inflating treatment effectiveness after treatment suspension. Descriptive and inferential statistical analyses were employed to summarize the cohort and assess differences between treatment groups. Continuous variables were expressed as means and standard deviations (SD).

Categorical variables were presented as absolute frequencies and/or percentages. Continuous variables were inspected for normality (Shapiro–Wilk test when n < 30). Between-group comparisons used the independent-samples t-test when assumptions were met. Otherwise, the Mann–Whitney U test was applied. Within-group changes over time were analyzed using the paired t-test (or the Wilcoxon signed-rank test when the data were not normally distributed). For longitudinal summaries, absolute change (*Δ*) scores between time points (e.g., Δw0–w16, Δw0–w24, Δw0–w52) were computed and compared between groups with the same criteria (independent-samples t or Mann–Whitney U) and within groups with paired tests. Categorical variables (e.g., responder rates such as EASI75/90/100, IGA 0/1, adverse event frequencies, and concomitant therapy use) were compared using Pearson’s χ^2^ test or Fisher’s exact test when expected cell counts were <5.

Associations between the number of prior therapies and outcomes (e.g., discontinuation, super-responder status) were explored using Spearman’s rank correlation (*ρ*). Drug survival was analyzed by comparing treatment duration (months) between groups as a continuous variable using an independent-samples t-test or the Mann–Whitney U test, as appropriate. All tests were two-sided, with statistical significance set at *p* < 0.05, and 95% confidence intervals (CIs) were reported where applicable. Results are presented as “(Group 1 vs. Group 2, *p*-value)” unless otherwise specified. Odds ratios (ORs) are reported with their 95% CI. Statistical analyses were performed with SPSS version 25.0 (IBM, Chicago, IL, USA). No formal multivariable adjustment (e.g., regression modeling or propensity score methods) or corrections for multiple testing were applied. Given the exploratory nature of this real-world clinical practice study and the available sample size, we considered that multiplicity adjustments would be overly conservative and might increase the risk of Type II errors. This limitation has been acknowledged and further discussed in the corresponding section.

During the manuscript preparation process, the authors utilized ChatGPT-4 (OpenAI, California, USA), under human oversight, to enhance the clarity and readability of certain sections. The final content was reviewed, edited, and fully approved by the authors, who take complete responsibility for its integrity.

### Ethical considerations

This study was conducted in accordance with the principles of the Declaration of Helsinki and received approval from the Institutional Review Board of Hospital Universitario San Cecilio, Granada, Spain (protocol code FIB-DAT-2024-20 v1.1; date of approval: October 31, 2024).

## Results

A total of 202 patients with moderate-to-severe AD were included in the study. Among them, 134 patients (66.3%) initiated treatment with biologic agents (Group 1), while 68 patients (33.7%) received JAKi (Group 2). Within the biologic group, the majority of patients were treated with dupilumab (*n* = 106, 79%), followed by tralokinumab (*n* = 20, 15%) and lebrikizumab (n = 8, 6%). In the JAKi group, upadacitinib was the most frequently prescribed agent (*n* = 35, 51%), followed by abrocitinib (*n* = 25, 37%) and baricitinib (*n* = 8, 12%).

Regarding the dosage regimen, the majority of patients in both groups received a standard (non-modified) posology throughout the entire treatment: 79.85% in the biologics group and 85.29% in the JAKi group. A smaller proportion of patients underwent posology optimization, either by extending the dosing interval or by reducing the administered dose depending on the drug (18.66% vs. 14.71%). The differences in the regimens used were not statistically significant between groups (*p* = 0.45273). It is noteworthy that the use of any concomitant systemic therapy—excluding short courses of corticosteroids—as adjuvant treatment during at least part of the period in which the AST was ongoing was significantly more frequent in the biologic group compared to the JAKi group. Specifically, 35.07% of patients treated with biologics required additional concomitant therapy, compared to only 10.29% among those receiving JAKi (*p* = 0.0001; OR 0.21 [0.09–0.50]) (Table 1).

**Table 1:** Sample distribution details of treatments studied.

Variable		Total	Group 1 (Biologics)	Group 2 (JAKi)	*p* value	Odds ratio (OR)
Sample size *n* (%)		202 (100%)	134 (66.34%)	68 (33.66%)	–	
			Dupi- 106 (79%)	Upa- 35 (51%)		
			Tralo- 20 (15%)	Abro- 25 (37%)		
			Lebri- 8 (6%)	Bari- 8 (12%)		
Posology *n* (%)	Standard	165 (81.68%)	107 (79.85%)	58 (85.29%)	0.45273	
	Optimized	35 (17.33%)	25 (18.66%)	10 (14.71%)		
	Intensified	2 (0.99%)	2 (1.93%)	0 (0.0%)		
Adjuvant therapy	No	148 (73.27%)	87 (64.93%)	61 (89.71%)	0.0001*	OR 0.21 (0.09–0.50)
	Yes	54 (26.73%)	47 (35.07%)	7 (10.29%)		

The standard posology regimen refers to the one approved as per the technical data sheet. Statistically significant comparisons are indicated in bold (*).

Baseline characteristics related to sociodemographics, lifestyle habits, and non-AD-related medical history—including cardiovascular risk factors—have already been analyzed in detail in a previous publication (54). In this study, these data are summarized again to reflect a slight increase in sample size; however, this expansion did not change the previously observed lack of statistical significance. In fact, the increase in sample size corrected the previously found difference in hypertension (10.45% vs. 7.35%, *p* = 0.64765). These results are summarized in Table 2. To this extent, no significant differences were found between treatment groups in terms of age at disease onset, duration of disease prior to treatment initiation, presence of disease-free periods during life, or reported seasonal variability in disease severity. The proportion of patients who required hospital admission for AD-related reasons, although higher clinically in the biologic group, was not significantly different between groups (10.45% vs. 2.94%, *p* = 0.1116). However, a significantly higher proportion of patients in the JAKi group reported prior ED visits due to AD flares (69.0.40% vs. 85.29%, *p* = 0.0223*).

**Table 2:** Baseline characteristics.

Category	Variable			Total		Group 1		Group 2		*p* value
Sociodemographics	Sample size		*n* (%)	202 (100%)		134 (66.34%)		68 (33.66%)		***-***
	Sex	Men	Absolut number	118		79		39		0.946
		Women		84		55		29		
		Ratio	Men: women	1.4		1.44		1.34		
	Age (years)		Mean ± SD	35.27 ± 14.81		34.99 ± 15.85		35.82 ± 12.60		0.6833
	Patients under 18		Absolut number	16		13		3		0.27175
	Body Mass Index (kg/m^2^)		Mean ± SD	24.93 ± 4.22		24.75 ± 4.54		25.33 ± 3.42		0.3388
	Abd. Per. (cm)	Men	Mean ± SD	92.98 ± 11.29		93.01 ± 11.40		92.91 ± 11.20		0.9659
		Women	Mean ± SD	80.41 ± 14.72		79.10 ± 15.69		83.25 ± 12.17		0.2136
Habits	Physical exercise (days per week)		Mean ± SD	2.36 ± 2.06		2.20 ± 1.97		2.70 ± 2.21		0.141
	Toxic habits	Any (present or past)	Absolut number	Yes	134	Yes	91	Yes	43	0.590
				No	59	No	37	No	22	
				Unknown	9	Unknown	6	Unknown	3	
Medical History	CVRF		Absolut number	Yes	46	Yes	34	Yes	12	0.313
				No	155	No	100	No	55	
				Unknown	1	Unknown	0	Unknown	1	
	Non-atopic comorbidities		Absolut number	Yes	117	Yes	76	Yes	41	0.737
				No	85	No	58	No	27	
				Unknown	0	Unknown	0	Unknown	0	
	Allergies		Absolut number	Yes	168	Yes	110	Yes	58	0.707
				No	34	No	24	No	10	
				Unknown	0	Unknown	0	Unknown	0	
	Atopic comorbidities		Absolut number	Yes	146	Yes	101	Yes	45	0.225
				No	56	No	33	No	23	
				Unknown	0	Unknown	0	Unknown	0	
	Family history of atopic dermatitis		Absolut number	Yes	101	Yes	70	Yes	31	0.457
				No	101	No	64	No	37	
				Unknown	0	Unknown	0	Unknown	0	
	Family history of atopic comorbidities (asthma, hay fever)		Absolut number	Yes	84	Yes	57	Yes	27	0.814
				No	118	No	77	No	41	
				Unknown	0	Unknown	0	Unknown	0	
Disease history	Age at debut		Mean ± SD	13.58 ± 16.81		12.89 ± 17.60		14.96 ± 15.16		0.3909
	Disease-free period in life		Number (%)	59 (29.21%)		44 (32.84%)		15 (22.06%)		0.1533
	Years of evolution pre-		Mean ± SD	20.34 ± 11.86		20.67 ± 12.43		19.69 ± 10.71		0.5616
	Visits to ED pre-		Number (%)	151 (74.75%)		93 (69.40%)		58 (85.29%)		0.0223*
	Admissions pre-		Number (%)	16 (7.92%)		14 (10.45%)		2 (2.94%)		0.1116
	Seasonal changes in disease severity		Number (%)	138 (68.31%)		90 (67.16%)		48 (70.59%)		0.978
Treatment history	Use of emolient (days per week)		Mean ± SD	4.90 ± 2.48		4.84 ± 2.50		5.01 ± 2.46		0.636
	Prior exposition to classic systemic therapies	Number (any)	Mean ± SD	1.78 ± 1.06		1.62 ± 0.96		2.09 ± 1.19		0.0058*
		CsA	Number (%)	188 (93.07%)		122 (91.04%)		66 (97.06%)		0.19454
		MTX		65 (32.18%)		38 (28.36%)		27 (39.71%)		0.141
		AZA		25 (12.38%)		12 (8.96%)		13 (19.12%)		0.06481
		MM		31 (15.35%)		16 (11.94%)		15 (22.06%)		0.09317
		UV		39 (19.31%)		39 (19.31%)		14 (20.59%)		0.88862
		IgIV		11 (5.45%)		4 (2.99%)		7 (10.29%)		0.06646
	Prior exposition to AST	Patients	*n* (%)	60 (29.70)		27 (20.15%)		33 (48.53%)		0.0001*
		Number (any)	Mean ± SD	0.36 ± 0.70		0.21 ± 0.49		0.66 ± 0.92		0.0003*
		Biologics	Mean ± SD	0.18 ± 0.43		0.08 ± 0.28		0.37 ± 0.60		0.0003*
		JAKi	Mean ± SD	0.18 ± 0.44		0.13 ± 0.33		0.29 ± 0.57		0.0291*

The table includes a summary of sociodemographic data, body measurements, lifestyle choices, and non-AD-related past medical history from participants, which have been extensively and more individually characterized in reference (53). Disease history and prior treatment exposure, stratified by treatment group, are more deeply analyzed. Mean ± standard deviation (SD) is given for continuous variables, and absolute numbers and/or percentages for categorical variables. Statistically significant comparisons are indicated in bold (*).

### Prior treatment history

No significant differences were observed between groups in the frequency of emollient use prior to initiation of advanced therapy (mean 4.90 ± 2.48 days/week overall; *p* = 0.636). When analyzed individually, there were no differences in the types of conventional classic therapies previously used by patients in both groups. However, patients in the biologics group had received a significantly lower number of prior conventional systemic therapies (cyclosporine A, methotrexate, azathioprine, mycophenolate mofetil, phototherapy, intravenous immunoglobulin) compared to those in the JAKi group (1.62 ± 0.96 vs. 2.09 ± 1.19, *p* = 0.0058*).

Previous exposure to AST was also more common in the JAKi group. Nearly half of the patients in this group (48.53%) had been treated with at least one prior advanced systemic agent, compared to only 20.15% in the biologic group (*p* = 0.0001*). Consequently, the mean number of prior targeted treatments was significantly lower in the biologics group (0.21 ± 0.49 vs. 0.66 ± 0.92, *p* = 0.0003*), with notable differences in prior exposure to both biologic therapies (0.08 ± 0.28 vs. 0.37 ± 0.60, *p* = 0.0003*) and JAKi (0.13 ± 0.33 vs. 0.29 ± 0.57, *p* = 0.0291*). Refer to Table 2.

### Efficacy summary: short (16 weeks) and medium-to-long-term (24 and 52 weeks)

Baseline severity scores were slightly different between groups. Patients in the JAKi group had significantly lower EASI and BSA scores at treatment initiation (*p* = 0.001* and *p* = 0.0007*, respectively), suggesting a slightly lower initial disease burden in this subgroup. At each of the subsequent assessment time points, data availability was as follows: in the biologics group, 125/134 patients (93.3%) at week 16, 112/134 (83.6%) at week 24, and 94/134 (70.1%) at week 52; in the JAKi group, 57/68 patients (83.8%) at week 16, 52/68 (76.5%) at week 24, and 38/68 (55.9%) at week 52. Missing data at later time points primarily reflected treatment discontinuation. A total of 10 patients in the biologic group and two patients in the JAKi group had no long-term data available, as they had not yet reached the corresponding time points at the time of analysis.

At week 16, significantly higher response rates were observed in the JAKi group across several key endpoints. EASI75 and EASI90 response rates were 73.7 and 57.9% in the JAKi group, compared to 56.6 and 36.9% in the biologic group (*p* = 0.0419* and *p* = 0.01317*, respectively).

Complete disease clearance (EASI100) at this time point was also more frequent in the JAKi group (36.8% vs. 19.7%, *p* = 0.02249; OR 0.41 [0.2–0.83]). Consistently, mean EASI, BSA, and IGA scores at week 16 were significantly, or almost significantly, lower in the JAKi group (*p* = 0.0035*, *p* = 0.0001*, and *p* = 0.07401, respectively). Differences in efficacy were attenuated by week 24, at which point response rates remained high in both groups. Although numerically greater for EASI75 and EASI90 in the JAKi group, these differences did not reach statistical significance. EASI100 was the only relative measure still significantly higher in the JAKi group (40.0% vs. 59.6%, *p* = 0.0301*) at this checkpoint. However, absolute measurements still favor JAKi over biologics. The EASI score (4.35 ± 8.76 vs. 1.60 ± 2.99, *p* = 0.0035*), BSA (4.08 ± 9.22 vs. 1.63 ± 3.42, *p* = 0.0149*), and IGA (0.82 ± 1.01 vs. 0.56 ± 0.80, *p* = 0.07401*) are significantly lower in this group.

**Figure 1:**
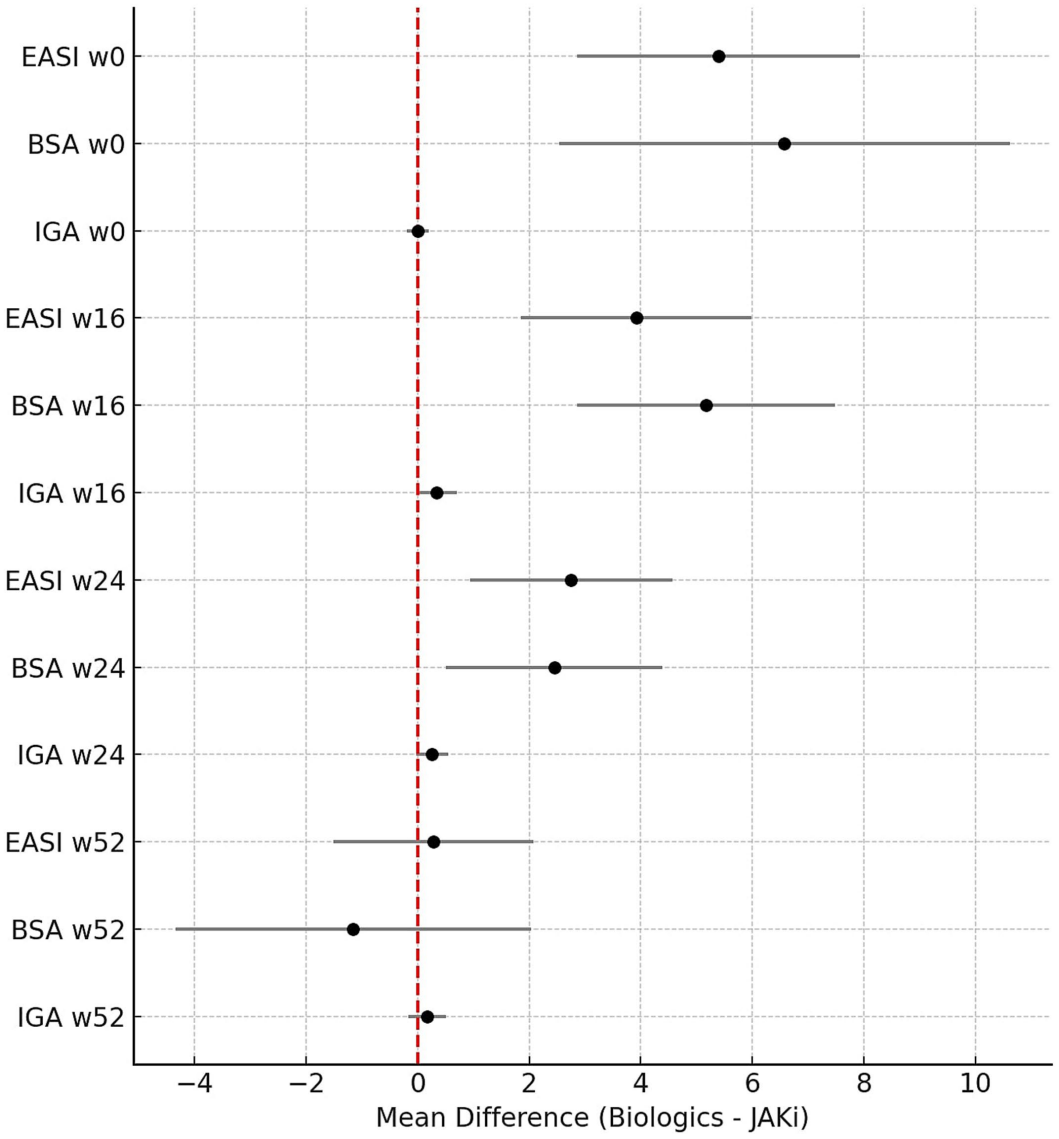
Forest plot showing the mean differences (Biologics – JAK inhibitors) with 95% confidence intervals for EASI, BSA, and IGA scores at baseline and at weeks 16, 24, and 52, as measures of effect size. Positive values indicate higher scores in the biologic group, whereas negative values indicate higher scores in the JAK inhibitor group.

At week 52, response rates continued to increase across both groups. Although the proportion of patients achieving EASI100 was still numerically lower in the biologics group (49.5% vs. 68.4%), the difference lost statistical significance (*p* = 0.0715) with an OR of 0.45 (0.2–1.0). Final EASI, IGA, and BSA scores at week 52, as well as VAS scores for pruritus, pain, and sleep disturbances post-treatment, did not differ significantly between the groups. A graphic representation of mean differences across time points for the main parameters (EASI, IGA, and BSA) is shown in Figure 1 to illustrate the magnitude and direction of the treatment effect across endpoints and time points. Patient satisfaction scores were also notably high in both cohorts and comparable (7.89 ± 2.87 vs. 8.05 ± 2.87, *p* = 0.357).

No statistically significant differences were observed between treatment groups regarding the proportion of those experiencing treatment-induced changes in clinical phenotype (47.6% vs. 33.82%, *p* = 0.0818). However, clinically, biologics appear to have a higher tendency to alter the presenting clinical picture, with an OR of 1.79 (0.98–3.28). Post-treatment ED visits were infrequent and comparable across groups (7.43% overall, *p* = 0.3789), with an overall reduction from 74.75 to 7.43% across the sample (Table 3).

**Table 3:** Efficacy scores, safety profile, treatment discontinuation, causes of failure (F), and drug survival analyses.

	Variable			Total	Group 1	Group 2	*p* value	Odds ratio (OR)
Treatment outcomes	VAS pruritus pre-		Mean ± SD	8.33 ± 2.22	8.45 ± 2.06	8.10 ± 2.50	0.3297	
	VAS pain pre-		Mean ± SD	6.08 ± 3.49	5.96 ± 3.51	6.32 ± 3.46	0.478	
	VAS sleep pre-		Mean ± SD	6.60 ± 3.5	6.85 ± 3.29	6.12 ± 3.90	0.187	
	EASI week 0		Mean ± SD	26,07 ± 9.66	27.89 ± 9.97	22.50 ± 7.96	0.0001*	
	BSA week 0		Mean ± SD	22.10 ± 15.33	24.42 ± 16.62	17.84 ± 12.18	0.0007*	
	IGA week 0		Mean ± SD	3.74 ± 0.70	3.74 ± 0.70	3.74 ± 0.68	0.97278	
	EASI week 16		Mean ± SD	6,36 ± 8,30	7,59 ± 9,19	3,67 ± 5,00	0.0003*	
	EASI50 w16		Percentage	84.9	83.6	87.7	0.62264	OR 0.65 (0.26–1.62)
	EASI75 w16		Percentage	62.0	56.6	73.7	0.04194*	**OR 0.45 (0.23–0.89)**
	EASI90 w16		Percentage	43.6	36.9	57.9	0.01317*	**OR 0.41 (0.22–0.79)**
	EASI 100 w16		Percentage	25.1	19.7	36.8	0.02249*	**OR 0.41 (0.2–0.83)**
	BSA week 16		Mean ± SD	6.02 ± 10.33	7.64 ± 11.85	2,47 ± 3,94	0.0001*	
	IGA week 16		Mean ± SD	1.25 ± 1.15	1.36 ± 1.15	1.02 ± 1.13	**0.06152**	
	EASI week 24		Mean ± SD	3.48 ± 7.53	4.35 ± 8.76	1,60 ± 2,99	0.0035*	
	EASI50 w24		Percentage	95.1	94.5	96.2	0.95794	OR 0.59 (0.12–2.96)
	EASI75 w24		Percentage	84.6	81.8	90.4	0.23955	OR 0.46 (0.16–1.29)
	EASI90 w24		Percentage	66.7	62.7	75.0	0.17116	OR 0.55 (0.26–1.14)
	EASI 100 w24		Percentage	46.3	40.0	59.6	0.0301*	**OR 0.44 (0.23–0.87)**
	BSA week 24		Mean ± SD	3.30 ± 7.93	4.08 ± 9.22	1.63 ± 3.42	0.0149*	
	IGA week 24		Mean ± SD	0.74 ± 0.95	0.82 ± 1.01	0.56 ± 0.80	**0.07401**	
	EASI week 52		Mean ± SD	2.15 ± 4.13	2.23 ± 3.68	1.95 ± 5.14	0.7554	
	EASI50 w52		Percentage	96.2	95.7	97.4	1	OR 0.6 (0.07–5.56)
	EASI75 w52		Percentage	90.8	92.5	86.8	0.49639	OR 1.86 (0.55–6.28)
	EASI90 w52		Percentage	75.6	74.2	78.9	0.72588	OR 0.77 (0.31–1.9)
	EASI 100 w52		Percentage	55.0	49.5	68.4	**0.0715**	**OR 0.45 (0.2–1.0)**
	BSA week 52		Mean ± SD	1.94 ± 5.71	1.60 ± 2.59	2.76 ± 9.89	0.4790	
	IGA week 52		Mean ± SD	0.59 ± 0.85	0.64 ± 0.81	0.47 ± 0.92	0.34114	
	VAS pruritus post-		Mean ± SD	2.80 ± 3,01	2.77 ± 2.91	2.88 ± 3,23	0.8329	
	VAS pain post-		Mean ± SD	1.45 ± 2.91	1.33 ± 2.79	1.71 ± 3.15	0.436	
	VAS sleep post-		Mean ± SD	1.44 ± 2.87	1.37 ± 2.76	1.59 ± 3.11	0.655	
	Satisfaction post-		Mean ± SD	7.89 ± 2.97	8.05 ± 2.87	7.57 ± 3.16	0.3573	
	Super-responders		*n* (%)	38 (18.81%)	22 (16.42%)	16 (23.53%)	0.25443	OR 0.638 (0.310–1.316)
	Phenotype change		*n* (%)	87 (43.07%)	64 (47.76%)	23 (33.82%)	**0.0818**	**OR 1.79** **(0.98–3.28)**
	Visits to ED post-		*n* (%)	15 (7.43%)	12 (8.96%)	3 (4.41%)	0.3789	OR 2.13 (0.58–7.82)
	Adverse events	Total	*n* (%)	91 (45.05%)	63 (47.01%)	28 (41.18%)	0.43062	OR 1.27 (0.7–2.29)
		Mild	*n* (%)	78 (38.61%)	54 (40.30%)	24 (35.30%)	0.48997	OR 1.24 (0.68–2.27)
		Moderate	*n* (%)	22 (10.89%)	13 (9.70%)	9 (13.24%)	0.44614	OR 0.7 (0.28–1.74)
		Severe	*n* (%)	2 (0.99%)	0 (0.00%)	2 (2.94%)	0.11221	OR 0.10 (0.01–2.09)
	Incidence of treatment suspension	Total	*n* (%)	58 (28.71%)	30 (22.39%)	28 (41.18%)	0.00867*	**OR 0.41 (0.22–0.77)**
		Primary F.	*n* (%)	17 (8.42%)	10 (7.46%)	7 (10.29%)	0.6768	OR 0.7(0.26–1.94)
		Secondary F.	*n* (%)	21 (10.40%)	8 (5.97%)	13 (19.12%)	0.0081*	**OR 0.27 (0.11–0.68)**
		Tolerance F.	*n* (%)	20 (9.90%)	14 (10.45%)	6 (8.82%)	0.9077	OR 1.21 (0.44–3.29)
		Patient D.	*n* (%)	9 (4.45%)	5 (3.73%)	4 (5.88%)	0.73432	OR 0.62 (0.16–2.39)
		Other	*n* (%)	2 (0.99%)	0 (0%)	2 (2.94%)	0.21378	OR 0.10 (0.00–2.09)
	Months of survival	Total	Mean ± SD	22.54 ± 18.71	26.62 ± 20.18	14.51 ± 11.99	0.00001*	
		Suspended (Mean ± SD)	All	14.52 ± 14.14	16.5 ± 16.88	12.39 ± 10.34	0.26604	
			Primary F.	3.82 ± 1.81	4.60 ± 1.96	2.71 ± 0.76	0.01655*	
			Secondary F.	15.76 ± 8.95	17.25 ± 9.63	14.85 ± 8.77	0.57496	
			Tolerance F.	11.25 ± 11.06	12.07 ± 12.4	9.33 ± 7.66	0.55675	
			Patient D.	25.78 ± 24.15	39.0 ± 25.08	9.25 ± 7.80	**0.05483**	
		Ongoing	Mean ± SD	25.78 ± 19.39	29.54 ± 20.18	16.00 ± 12.94	0.00001*	

Mean ± standard deviation is provided for continuous variables, and absolute numbers and/or percentages for categorical variables. Statistically significant comparisons are highlighted in bold (*). Some additional relevant results close to statistical significance are also highlighted in bold (without *). Odds ratios (OR) with 95% confidence intervals (CI) are provided where appropriate.

#### Super-responders

A total of 38 patients (18.81%) were classified as super-responders, including 22 (16.42%) from the biologic group and 16 (23.53%) from the JAKi group (*p* = 0.25443, OR 0.638 [0.310–1.316]). This subgroup represents a clinically significant portion of patients who achieve deep and sustained responses with optimal tolerability without treatment intensification or adjunctive therapy, as defined in the Methods section (Table 3).

#### Extensive effectiveness characterization: magnitude of change

The magnitude of change in disease severity scores has been described in detail. Improvements in VAS scores for pruritus, pain, and sleep disturbance from baseline to post-treatment were substantial in both groups, with no statistically significant differences observed in the magnitude of change. In contrast, changes in EASI and BSA scores revealed several significant differences favoring the biologic group in terms of absolute improvements. From baseline to week 24 and baseline to week 52, the mean change in EASI score was greater in the biologic group compared to the JAKi group (*p* = 0.00923* and *p* = 0.00014*, respectively). Similarly, EASI improvement from week 16 to week 52 was still significantly greater in the biologic group (*p* = 0.00107*). Notably, there appears to be a lack of improvement in severity, considering the score changes in the JAKi group between weeks 24 and 52 (with a mean increase in EASI of 0.67 ± 5.49 and a mean increase in BSA of 1.21 ± 10.34). However, we must consider that in this treatment group, severity scores had already decreased by week 24 (mean EASI = 1.60 ± 2.99 and mean BSA = 1.63 ± 3.42), as analyzed in the previous section. For BSA, the magnitude of change from baseline to week 24 and baseline to week 52 was significantly higher in the biologic group (*p* = 0.00945* and *p* = 0.00268*, respectively). This is mainly due to differences specifically within the periods from week 16 to week 24 and from week 16 to week 52 (*p* = 0.00682* and *p* = 0.00187*, respectively), when the full effect of biologic drugs is expected to materialize. The percentage of improvement from baseline to week 52 in EASI, BSA, and IGA was high in both groups and did not differ significantly, indicating the overall effectiveness of both treatment classes, despite distinct dynamics in the evolution of responses. All the intergroup comparisons of absolute and relative changes in severity score are shown in Table 4.

**Table 4:** Intergroup comparisons of the magnitude of change in disease severity scores, expressed as absolute and relative decreases.

Variable		Group 1	Group 2	*p* value
∇Change in VAS pruritus pre- > post	Mean ± SD	5.82 ± 3.39	5.34 ± 3.76	0.42019
∇Change in VAS pain pre- > post	Mean ± SD	4.56 ± 4.15	4.88 ± 3.90	0.62820
∇Change in VAS sleep pre- > post	Mean ± SD	5.51 ± 4.14	4.86 ± 4.08	0.33114
∇ Change in EASI w0- > w16	Mean ± SD	20.7 ± 12.3	18.0 ± 8.94	0.09703
∇ Change in EASI w0- > w24	Mean ± SD	24.65 ± 13.5	20.07 ± 8.47	0.00923*
∇ Change in EASI w0- > w52	Mean ± SD	27.91 ± 10.44	20.71 ± 8.85	0.00014*
(%∇) Change in EASI w0- > w52	Mean ± SD	91.57 ± 15.02	90.91 ± 21.71	0.86355
∇ Change in EASI w16- > w24	Mean ± SD	2.6 ± 9.93	1.32 ± 3.08	0.21578
∇ Change in EASI w16- > w52	Mean ± SD	4.25 ± 8.47	0.08 ± 5.42	0.00107*
∇ Change in EASI w24- > w52	Mean ± SD	0.86 ± 4.29	−0.67 ± 5.49	0.12827
∇ Change in BSA w0- > w16	Mean ± SD	17.33 ± 15.91	14.25 ± 11.2	0.13783
∇ Change in BSA w0- > w24	Mean ± SD	21.16 ± 17.35	15.06 ± 11.72	0.00945*
∇ Change in BSA w0- > w52	Mean ± SD	24.81 ± 17.22	15.26 ± 15.4	0.00268*
(%∇) Change in BSA w0- > w52	Mean ± SD	92.3 ± 13.48	82.07 ± 69.78	0.37545
∇ Change in BSA w16- > w24	Mean ± SD	2.75 ± 8.83	0.19 ± 3.03	0.00682*
∇ Change in BSA w16- > w52	Mean ± SD	4.65 ± 9.81	−1.5 ± 9.92	0.00187*
∇ Change in BSA w24- > w52	Mean ± SD	1.22 ± 5.02	−1.21 ± 10.34	0.17266
∇ Change in IGA w0- > w16	Mean ± SD	2.38 ± 1.27	2.68 ± 1.38	0.16606
∇ Change in IGA w0- > w24	Mean ± SD	2.96 ± 1.14	3.13 ± 1.1	0.36453
∇ Change in IGA w0- > w52	Mean ± SD	3.19 ± 0.96	3.32 ± 1.07	0.53613
(%∇) Change in IGA w0- > w52	Mean ± SD	82.62 ± 22.69	87.28 ± 23.94	0.30827
∇ Change in IGA w16- > w24	Mean ± SD	0.43 ± 0.96	0.35 ± 0.86	0.58298
∇ Change in IGA w16- > w52	Mean ± SD	0.55 ± 1.06	0.26 ± 1.31	0.22981
∇ Change in IGA w24- > w52	Mean ± SD	0.09 ± 0.94	0.03 ± 0.94	0.7463

Negative values of reductions in these scores reflect an increase in disease severity. Statistically significant comparisons are highlighted in bold (*).

### Safety: AE

AEs were reported in 45.05% of patients. The majority of the patients affected (78 out of 91, 85.71%) were considered to have mild reactions. The overall distribution by severity did not differ significantly between groups. Severe AEs were rare, affecting only two patients in the JAKi group (0.99% of the total sample, 2.94% of the JAKi group, intergroup *p* = 0.11221).

Treatment discontinuation occurred in 28.71% of the total cohort, with significantly higher rates in the JAKi group (22.39% vs. 41.18%, *p* = 0.00867*; OR 0.41 [0.22–0.77]). Among the reasons for suspension, secondary failure was the most discriminative, being more common in patients treated with JAKi (6.0% vs. 19.1%, *p* = 0.0081*; OR 0.27 [0.11–0.68]). No significant differences were found for primary failure, tolerance issues, or patient-driven decisions. Parallelly, the overall treatment survival mean in months was significantly higher in the biologic group compared to the JAKi group (26.62 ± 20.18 vs. 14.51 ± 11.99, *p* = 0.00001*). When examining suspended treatments alone, the mean duration of use prior to discontinuation was shorter in the JAKi group (16.5 ± 16.88 months vs. 12.39 ± 10.34 months, *p* = 0.2604), with a statistically significant shorter survival among JAKi patients who discontinued due to primary failure (4.60 ± 1.96 months vs. 2.71 ± 0.76 months, *p* = 0.01655*). Moreover, treatment was suspended due to a patient-driven decision that appeared to occur much earlier during the months of treatment with JAKi (39.0 ± 25.08 vs. 9.25 ± 7.80 months, *p* = 0.05483). However, it did not reach statistical significance. Ongoing treatments at the time of analysis showed a mean survival of 25.78 ± 19.39 months overall, with longer ongoing persistence in the biologic group (29.54 ± 20.18 vs. 16.00 ± 12.94 months, *p* = 0.00001*) (Table 3).

#### Extensive AE characterization: individual patient-reported events

Reported AEs were analyzed by type and involved system. A broad spectrum of manifestations was recorded, encompassing dermatologic, ophthalmologic, infectious, gastrointestinal, systemic, neurological, and other conditions.

Facial erythema was clinically more frequent in the biologic group, although this difference did not reach statistical significance (*p* = 0.36234). The only cutaneous manifestation that exceeded the significance threshold was acne, which was significantly more frequent in the JAKi group (2 patients [6.72%] vs. 9 patients [13.24%], *p* = 0.00165*, OR 0.10 [0.02–0.47]). Ophthalmologic involvement was significantly more common in the biologic group (34 patients [25.37%] vs. five patients [7.35%], *p* = 0.00401*, OR 4.28, [1.59–11.53]), particularly among patients without any prior ophthalmologic history (*p* = 0.0066*). The most frequently reported symptoms were dry eye and pruritus, which were significantly more prevalent in the biologic group (*p* = 0.0014*; OR 12.45 [1.64–94.68]). Patients with infectious complications were more frequently found in the JAKi group (6 patients [4.48%] vs. 11 patients [16.18%], *p* = 0.01041*; OR 0.24 [0.09–0.69]), primarily due to respiratory infections (*p* = 0.00391*).

No statistically significant differences were found in herpesvirus-related reactivations or other viral infections; however, it is worth noting that the only patient with shingles was reported in the JAKi group. Gastrointestinal AEs were significantly more frequent in the JAKi group (one patient [0.75%] vs. five patients [7.35%], *p* = 0.02961*; OR 0.09 [0.01–0.83]). Systemic symptoms were numerically more frequent also in the JAKi group, although the difference did not reach statistical significance overall (*p* = 0.05804). No relevant differences were observed for locomotor or neurological symptoms. Only one case of thromboembolic events was reported (bilateral superficial saphenous vein thrombophlebitis) in a man from the JAKi group with no other known risk factors, including other medications, immobilization, major surgery, or prior thromboembolic disease (Table 5).

**Table 5:** Distribution of adverse events by type and organ system.

Variable		Total	Group 1	Group 2	*p* value	Odds ratio (OR)
Administration reaction	Local reaction	1	1	0	–	
	Systemic reaction	3	2	1	1	
Dermatologic rash/disease	Total (patients w/ events)	70	48	22	0.6428	OR 1.167 (0.63–2.17)
	Facial erythema	22	17	5	0.36234	OR 1.83 (0.65–5.20)
	Skin erythema (worsening)	8	6	2	0.88282	
	Skin xerosis (worsening)	3	3	0	0.53023	
	Skin itch (worsening)	5	3	2	1	
	Psoriasis	3	2	1	1	
	Lichenoid reaction	1	1	0	1	
	Urticaria	5	3	2	1	
	Seborrheic derm	5	5	0	0.25687	
	Acne	11	2	9	0.00165*	**OR 0.10** **(0.02–0.47)**
	Rosacea	1	1	0	1	
	Hair loss	6	5	1	0.64847	
Ophthalmologic involvement	Total (patients w/ events)	39	34	5	0.00401*	**OR 4.28** **(1.59–11.53)**
	With PH opht. dis.	13	11	2	**0.4120**	OR 2.75 (0.46–16.59)
	Without PH opth. Dis.	26	23	3	0.0066*	**OR 5.02** **(1.44–17.51)**
	Dry eye and itchiness	22	21	1	0.0014*	**OR 12.45 (1.64–94.68)**
	Conjunctivitis (non-HSV)	12	11	1	**0.06353**	OR 5.99 (0.76–47.42)
	Blepharitis/blepharospasm	2	2	0	0.55116	OR 2.06 (0.09–46.33)
	HSV infection/react.	5	2	3	0.33754	OR 0.33 (0.05–2.01)
	Foreign body sens.	1	1	0	1	OR 1.02 (0.03–30.86)
	Photophobia	1	1	0	1	OR 1.02 (0.03–30.86)
	Red eye (NOS)	2	2	0	0.55116	OR 2.06 (0.09–46.33)
Infections	Total (patients w/ events)	17	6	11	0.01041*	**OR 0.24 (0.09–0.69)**
	Respiratory	5	0	5	0.00391*	**OR 0.05** **(0.0–0.87)**
	Cutaneous	3	1	2	0.26257	OR 0.25 (0.02–2.79)
	HSV infection/react.	9	4	5	0.16742	OR 0.39 (0.1–1.49)
	Shingles	1	0	1	0.33663	OR 0.25 (0.01–7.55)
	Other/non-specified	1	1	0	1	OR 1.02 (0.03–30.86)
Locomotor symptoms	Total (patients w/ events)	6	3	3	0.67365	
	Myalgias	1	0	1	0.33663	OR 0.25 (0.01–7.55)
	Arthralgias	5	3	2	1	OR 0.76 (0.12–4.63)
GI symptoms	Total (patients w/events)	6	1	5	0.02961*	**OR 0.09** **(0.01–0.83)**
Systemic symptoms	Total (patients w/ events)	13	5	8	**0.05804**	**OR 0.29** **(0.09–0.93)**
	Syncopes and hypotension	3	1	2	0.26257	OR 0.25 (0.02–2.79)
	Asthenia	8	4	4	0.4463	OR 0.49 (0.12–2.03)
	Fever	1	1	0	1	OR 1.02 (0.03–30.86)
	Cholesterol increase	2	0	2	0.11221	OR 0.12 (0.01–2.77)
Neurological symptoms	Total (patients w/ events)	7	4	3	0.68974	OR 0.67 (0.14–3.07)
	Headache	6	4	2	1	
	Parestesia	1	0	1	0.33663	
Others	Total (patients w/ events)	7	1	6	0.00638*	**OR 0.08** **(0.01–0.66)**
	Hypothymia	2	1	1	1	OR 0.5 (0.03–8.18)
	Thromboembolic	1	0	1	0.33663	OR 0.25 (0.01–7.55)

Statistically significant comparisons are shown in bold (*). Some additional relevant results close to statistical significance are also in bold (without *). Odds ratios (OR) with 95% confidence intervals (CI) are included where appropriate. GI, gastrointestinal; HSV, herpes simplex virus; PH, previous history; NOS, not otherwise specified.

## Discussion

This study offers a robust analysis of RWD from two well-characterized and comparable cohorts of patients from a single healthcare area in the south of Spain with moderate-to-severe AD treated with biologics or JAKi. One of the key findings of the prior study that enabled this one was the confirmation that biologics and JAKi are not being preferentially prescribed to distinct patient populations in real-life practice in our area, in terms of sociodemographic data, lifestyle habits, and non-AD-related medical history—including cardiovascular risk factors—contrary to what some theoretical frameworks and early clinical assumptions might suggest (54). Building on the equivalence between the two groups in this study, the analysis delves into disease-related variables and treatment outcomes, providing one of the most comprehensive comparative assessments to date in routine clinical settings.

Patients were generally comparable across almost all variables related to their disease background. The main differences were ED visits prior to treatment initiation and, importantly, baseline EASI and BSA severity scores. However, despite these differences, the average patient in both cohorts was still considered equally severe, according to the EASI score (range: 22–72), and moderate, according to the BSA score (range: 10–50%), for both groups.

Regarding their prior treatment history, although patients in the JAKi group had received a significantly greater number of prior conventional systemic therapies, the clinical relevance of this difference is likely limited, as it represents fewer than 0.5 treatments on average, and there was no difference in the pattern of previous conventional therapies prescribed between groups. The most prescribed drug in this category was cyclosporine A (91.04% of patients in the biologic group and 97.06% in the JAKi group had received it), and a retrospective observational study showed that the duration and cumulative dose of prior cyclosporine treatment do not negatively affect the response to biologics or JAK inhibitors (60). The observed difference likely reflects the greater cumulative clinical experience with biologics in our region, which led to the earlier initiation of these agents and the deferred use of JAK inhibitors. Additionally, patients unwilling to self-inject had to wait longer for JAKi to become available. A more relevant factor, however, might be the percentage of patients who had previously been exposed to AST prior to treatment initiation, which was higher in the JAKi group. The absolute difference in the number of AST received remains very low, averaging around 0.4 therapies, which is likely attributable to the later availability of JAK inhibitors in real clinical practice. Whether this could potentially affect efficacy outcomes in this group of our cohort is being further explored in a separate ongoing analysis (35, 61). Overall, this pattern could indicate a marginally more refractory disease course in the JAKi group—characterized by a lack of complete control following previous treatments—which could partially explain their slightly lower baseline severity (as measured by EASI and BSA). However, these differences were quantitatively small and were carefully considered in the interpretation of treatment outcomes.

Regarding treatment persistence and drug survival, several important differences emerged. The duration of ongoing treatment was significantly longer among patients on biologics, which likely reflects not only the favorable long-term tolerability of biologics but also their earlier availability and wider clinical experience in routine practice. Interestingly, treatment discontinuation due to primary failure occurred earlier in the JAKi group, likely reflecting the more rapid onset of action of JAKi and, consequently, earlier evaluation and recognition of therapeutic failure (62). The different times to clinical response between these two families of treatments could also have influenced the differences found regarding the use of concomitant systemic treatments (excluding short corticosteroid courses), which were significantly higher in the biologic group. Although this could suggest a lower need for therapeutic supplementation among patients treated with JAKi, it is important to interpret this finding in context. As adjuvant therapies could have been used at any time during the treatment period, we should consider that many patients starting biologics may have used temporary bridging therapy during the latency period before achieving the complete clinical effect of the drug studied, which is typically longer for monoclonal antibodies compared to JAKi. Therefore, this statistical difference reflects routine clinical practice rather than a true increased need for concomitant therapies for long-term disease control.

Both therapeutic classes showed high efficacy rates and significant improvements in severity scales. Baseline severity—slightly higher in biologics—taken together with absolute and relative scores over time points to both similarities and nuanced differences between biologics and JAKi in our cohort. Whereas the maximum efficacy of JAKi is reached earlier in time, as previously suggested by prior studies (52), biologics catch up by week 24, with only remaining differences in their respective EASI100 scores, which also fade by week 52. Notably, the higher rates of relative EASI achieved by JAKi at week 16 occurred alongside a not statistically significant higher absolute reduction in EASI and BSA scores in the biologics group between weeks 0 and 16, which would be the parameter that could theoretically be influenced by higher baseline values. This pattern is consistent with pharmacodynamic differences between drug classes. From baseline to week 24 and baseline to week 52, however, the mean absolute improvement in EASI was significantly greater in the biologic group. This was primarily driven by larger EASI reductions between weeks 16 and 52, a trend that aligns with the longer time to maximal effect reported for monoclonal antibodies. Therefore, although baseline EASI scores were slightly higher in the biologics group, the magnitude and timing of change are coherent with established pharmacological profiles and do not suggest distortion by initial imbalances. Furthermore, the percentage reduction in EASI, BSA, and IGA scores from baseline to week 52 did not differ significantly between groups, reinforcing the idea that both classes can achieve optimal responses despite minor baseline differences. Considering this information, we believe that therapeutic goals and existing algorithms in the literature for evaluating treatment outcomes over time should be adapted according to the type of AST used (63).

Notably, we have also found differences in their safety profile. While the present study confirms previously reported safety profiles of both treatment classes, it also offers new insights into their differential tolerability in real-life conditions. Overall, AE rates and AE severity profiles were comparable; however, specific differences emerged by organ system. Dermatologic AE—particularly facial erythema and xerosis—were more frequently reported in the biologic group, although not significantly. In contrast, acne was significantly more prevalent among patients treated with JAKi, a finding that has been previously associated with their broader immunomodulatory activity (50, 64, 65). Ophthalmologic complications, particularly dry eye and pruritus, were significantly more common in the biologic group, even among patients without prior ophthalmologic history. This may reflect the well-recognized alterations in ocular surface homeostasis observed with IL-4/IL-13 blockade (50).

Infectious events, particularly respiratory tract infections, were more frequent in the JAKi group, consistent with a known class-related, broader immunosuppressive profile. Gastrointestinal symptoms were also significantly more common among JAKi-treated patients, although they were mild. Other categories of AEs studied showed nil-to-minor differences, with most remaining non-significant. Notably, treatment discontinuation due to secondary failure was significantly higher in the JAKi group, whereas rates of primary failure, tolerance-related withdrawal, or patient decision did not differ between groups. These patterns, together with the shorter treatment survival in the JAKi group and their rapid onset of action, suggest that treatment decisions—and subsequent discontinuations—are made earlier in the JAKi treatment course, often based on early response assessments. This highlights the need to further refine follow-up strategies and response thresholds tailored to the pharmacodynamics of each therapeutic class.

## Strengths, limitations, and implications

This study presents multiple strengths that enhance both the validity and applicability of its findings. It is based on a large, well-characterized cohort of patients treated within a unified public healthcare system, ensuring standardized access criteria and treatment protocols. Its real-world design captures the complexity and heterogeneity of patients encountered in routine clinical dermatology, extending beyond the controlled conditions of RCTs. Given the ambispective nature of the study, the potential for missing data or unmeasured confounding variables in retrospective searches was addressed by using restrictive inclusion criteria, ensuring that the analysis was performed only on patients with sufficient baseline information.

Importantly, a prior in-depth analysis of baseline sociodemographic and medical history variables confirmed a high degree of comparability between the biologic and JAKi groups, enabling robust and unbiased comparisons of disease-specific characteristics and treatment outcomes. Additionally, the fact that patients were managed by a limited and consistent group of dermatologists within the same healthcare area minimizes inter-physician and institutional variability, thereby reducing potential confounding due to differences in clinical practice. Another notable strength lies in the systematic and comprehensive assessment of key variables, including disease severity indices, treatment response dynamics, AE profiles, drug survival, and patient-reported outcomes. This holistic evaluation provides a comprehensive understanding of therapeutic performance across multiple clinically relevant dimensions. Moreover, the geographically focused nature of the study, while potentially limiting generalizability, serves as a methodological advantage in the context of ongoing translational research by providing a controlled setting in which advanced therapies can be compared under real-world conditions in a single population and with minimal external variation.

Nevertheless, the study has inherent limitations. As with all observational designs, residual confounding cannot be entirely excluded, despite careful cohort characterization. After treatment suspension, no imputation was applied, which reduced the number of evaluable cases at later time points. However, this approach was considered preferable to methods such as last observation carried forward, which could have artificially overestimated treatment effectiveness in patients who discontinued due to insufficient response or adverse events. The choice of therapy was not randomized and may have been influenced by evolving drug availability or regulatory constraints, as addressed in previous sections. Additionally, the relatively shorter follow-up period for recently introduced agents, such as lebrikizumab and baricitinib, may have influenced their respective families’ grouped data regarding ongoing drug survival estimations. Furthermore, while strict operational definitions were applied to identify super-responders and treatment failure, the absence of universally accepted criteria for these constructs poses challenges for cross-study comparisons. Finally, patient-reported measures such as VAS and treatment satisfaction, although clinically informative, are inherently subjective and may be influenced by factors beyond disease control.

The study reinforces the value of conducting direct comparisons within well-balanced cohorts and provides further evidence to guide personalized treatment decisions in clinical practice.

## Conclusion

This real-world, ambispective study provides a comprehensive, comparative analysis of biologic agents and JAKi in the treatment of moderate-to-severe AD within a large, well-defined patient cohort. By ensuring baseline comparability and assessing a wide range of clinical, safety, and treatment-related outcomes, the study offers robust insights into the differential performance of these advanced therapies in routine clinical practice. Both therapeutic classes demonstrated high effectiveness and favorable safety profiles. JAKi was associated with a more rapid onset of response, whereas biologics showed more sustained absolute improvements over time and longer treatment persistence.

## Data Availability

The raw data supporting the conclusions of this article will be made available by the authors, without undue reservation.
